# Correction: Metformin inhibits the proliferation of benign prostatic epithelial cells

**DOI:** 10.1371/journal.pone.0295893

**Published:** 2023-12-12

**Authors:** Zongwei Wang, Xingyuan Xiao, Rongbin Ge, Jijun Li, Cameron W. Johnson, Cyrus Rassoulian, Aria F. Olumi

After publication of this article [1], concerns were raised about Figs 3 and 4. Specifically:

- In Fig 3D, the IGF-1 0ng/mL Met 5mM and IGF-1 100ng/mL Met 5mM panels overlapped with regions found in the PC3 Met/Mock and PC3 Met/M5 panels in Fig. 2M of [2].
- In Fig 4A, there appeared to be a vertical discontinuity between lanes 6 and 7 in the GAPDH panel.

**Fig 3:**
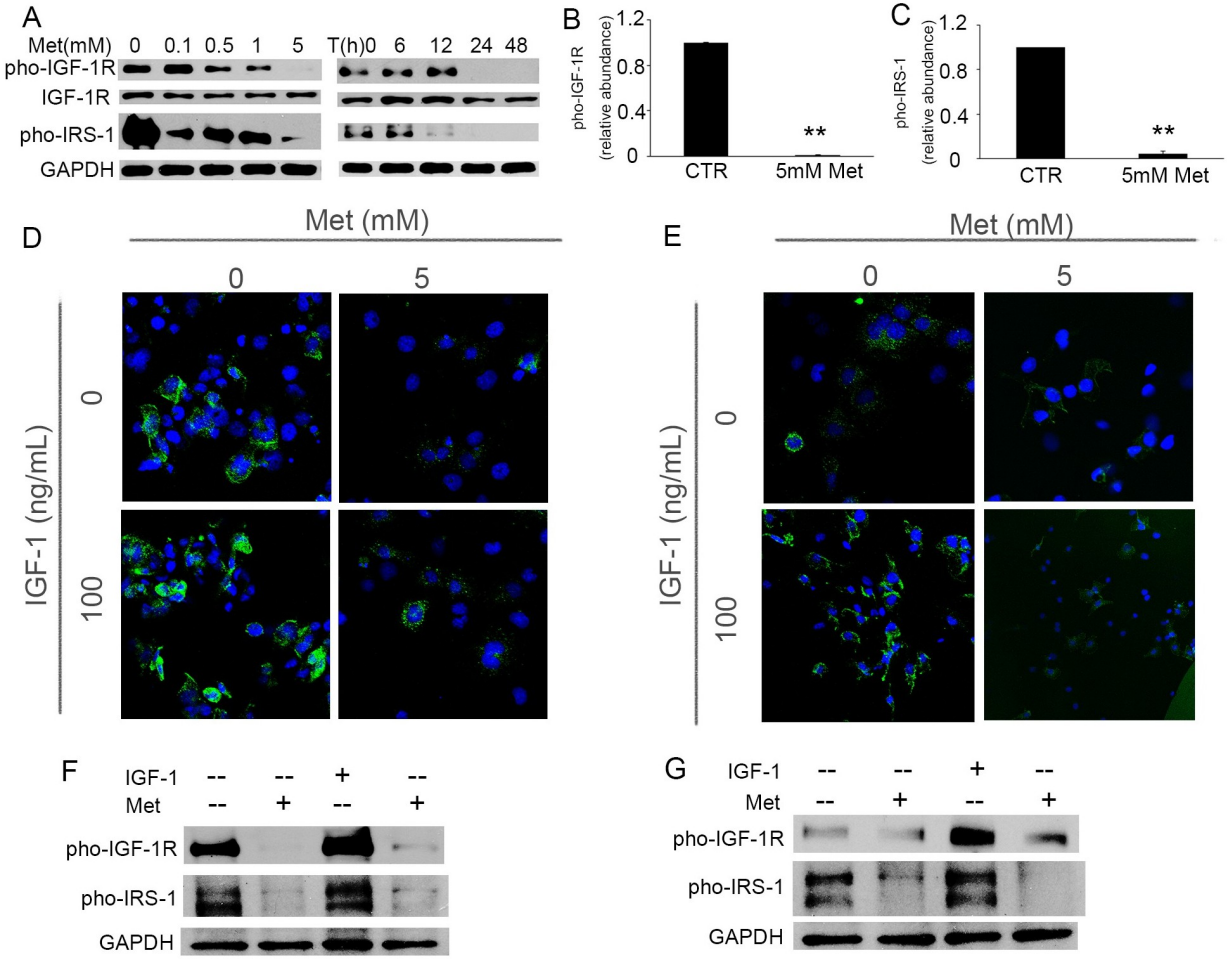
Metformin inhibits expression of IGF-1 receptor (IGF-1R) and abrogates IGF-1-induced phosphorylation of IGF-1R. A: Immunoblotting of IGF-1R, pho-IGF-1R and pho-IRS-1 in proliferating BPH-1 cells treated with various doses of metformin for 24 hours, or with 5 mM metformin for the indicated times. B-C: Quantification of the immunoblot protein expression of pho-IGF-1R (B) and pho-IRS-1 (C) in BPH-1 cells treated with 5 mM Metformin for 24 hours, and normalized to IGF-1R or GAPDH, respectively. **, *P*<0.001 compared with control group. D-E: Representative immunofluorescence images of pho-IGF-1R in BPH-1 (D) and P69 (E) cells after exposure to 100 ng/mL IGF-1 in the presence or absence of 5 mM metformin for 48 hours. F-G: Immunoblotting of pho-IGF-1R and pho-IRS-1 in BPH-1 (F) and P69 (G) cells 48 hours after treatment of IGF-1(100 ng/mL) and metformin (5 mM). CTR: untreated control, Met: metformin.]

**Fig 4:**
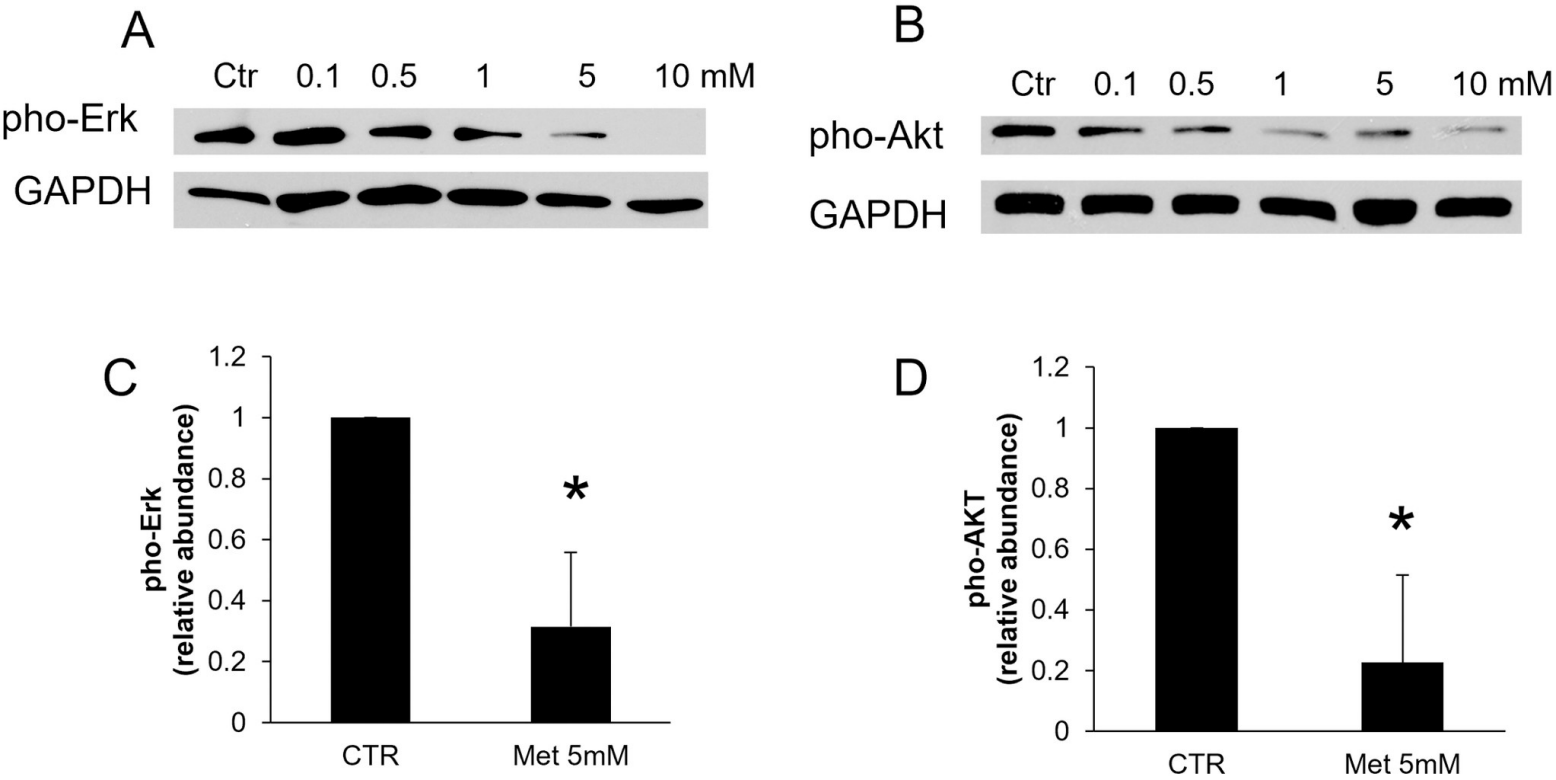
Metformin inhibits the expression of cell proliferating regulators, Erk and Akt. A-B: Representative immunoblot of three different experiments of pho-Erk (A) and pho-Akt protein levels (B) in BPH-1 cells 48h after treatment of metformin. C-D: Quantification of the immunoblot protein levels of pho-Erk (C) and pho-Akt (D) in BPH-1 cells treated with 5 mM metformin for 48 hours by normalization to GAPDH. *P*<0.05, compared with control group. CTR: control, Met: metformin.

Regarding Fig 3D, the corresponding author stated this was due to an error made in preparation of the figures and provided the original underlying data for this panel from the original experiments.

Regarding Fig 4A, the corresponding author stated that this panel was cropped imprecisely and provided the original underlying data and a panel with corrected cropping from the original experiments.

The corresponding author additionally stated that the AKT panel in Fig 4B was mislabeled and should have been labeled “GAPDH”, and provided the underlying data for this panel from the original experiments.

In addition, the primary data underlying results in this article were not included with the published article, although the Data Availability Statement for this article declared that all relevant data are within the paper.

With this Correction, the authors provide corrected panels for Figs 3 and 4, the original raw data for Fig 3 (S1 and S2 Files), and Fig 4 (S3 and S4 Files).

The authors apologize for the errors in the published article.

## Supporting information

S1 File Quantification data for Fig 3B and 3C. (XLSX)

S2 File Underlying data for Fig 3A, 3D, 3E, 3F and 3G. (PPTX)

S3 File Quantification data for Fig 4C and 4D. (XLSX)

S4 File Underlying data for Fig 4A and 4B. (TIF)
