# Supplementary material for: Correction: Metformin inhibits the proliferation of benign prostatic epithelial cells
Source: PLoS One. 2023 Dec 12;18(12):e0295893. doi: 10.1371/journal.pone.0295893 (PMC10715638; doi:10.1371/journal.pone.0295893)
Supplement: S2 File — (PPTX) [file pone.0295893.s002.pptx]

## Slide 1
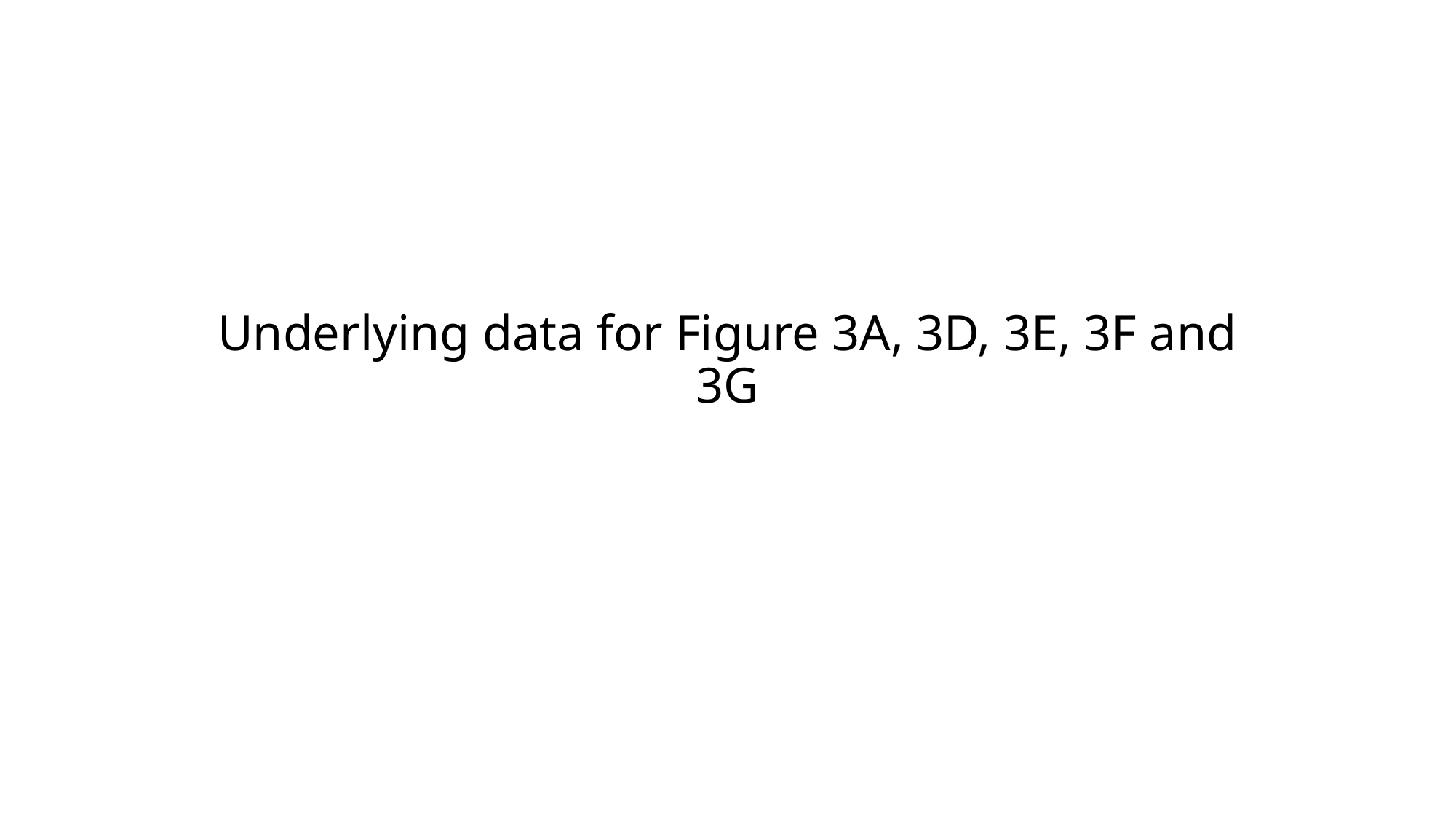

# Underlying data for Figure 3A, 3D, 3E, 3F and 3G

## Slide 2
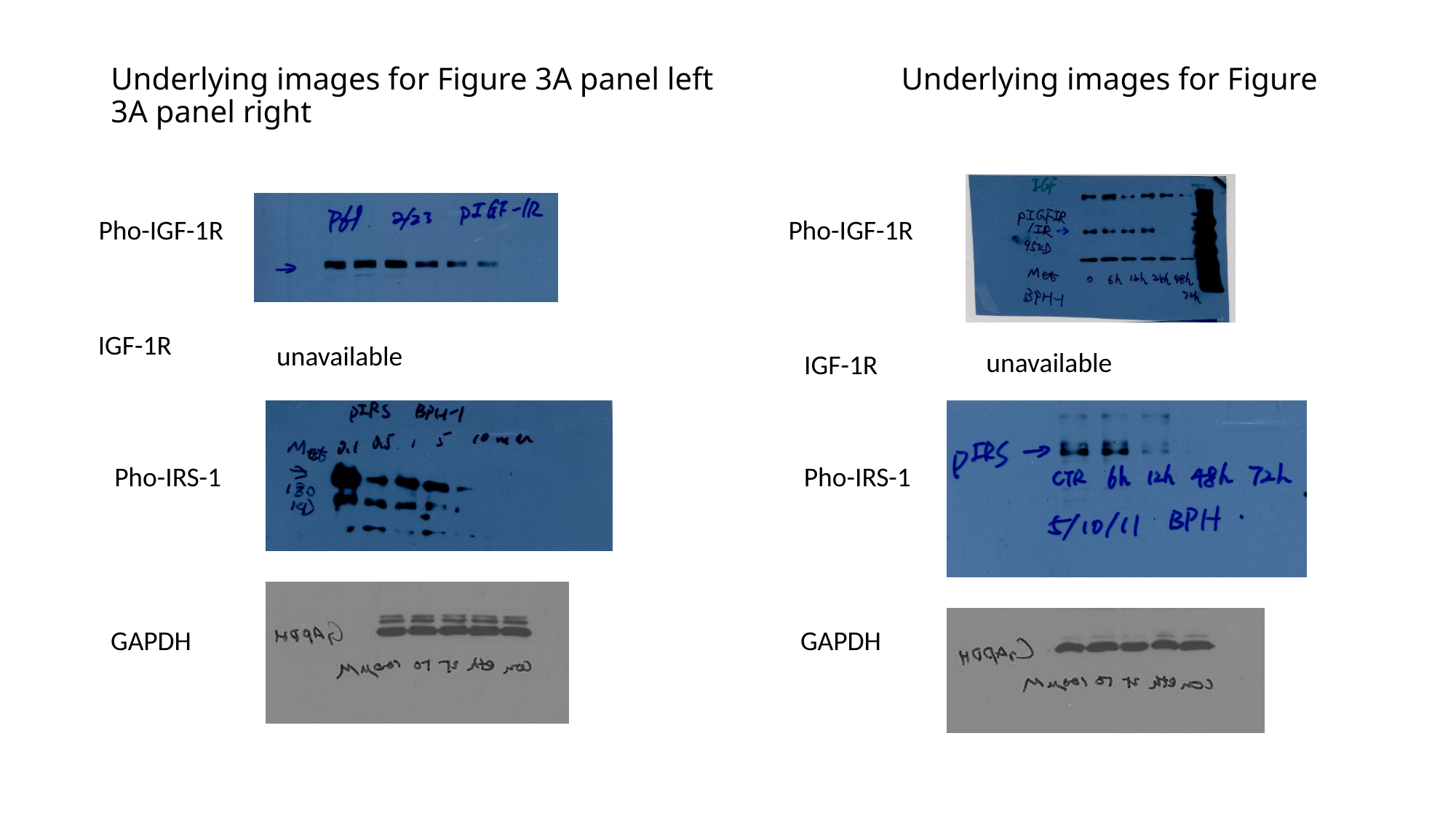

# Underlying images for Figure 3A panel left Underlying images for Figure 3A panel right
Pho-IGF-1R
Pho-IGF-1R
IGF-1R
unavailable
unavailable
IGF-1R
Pho-IRS-1
Pho-IRS-1
GAPDH
GAPDH

## Slide 3
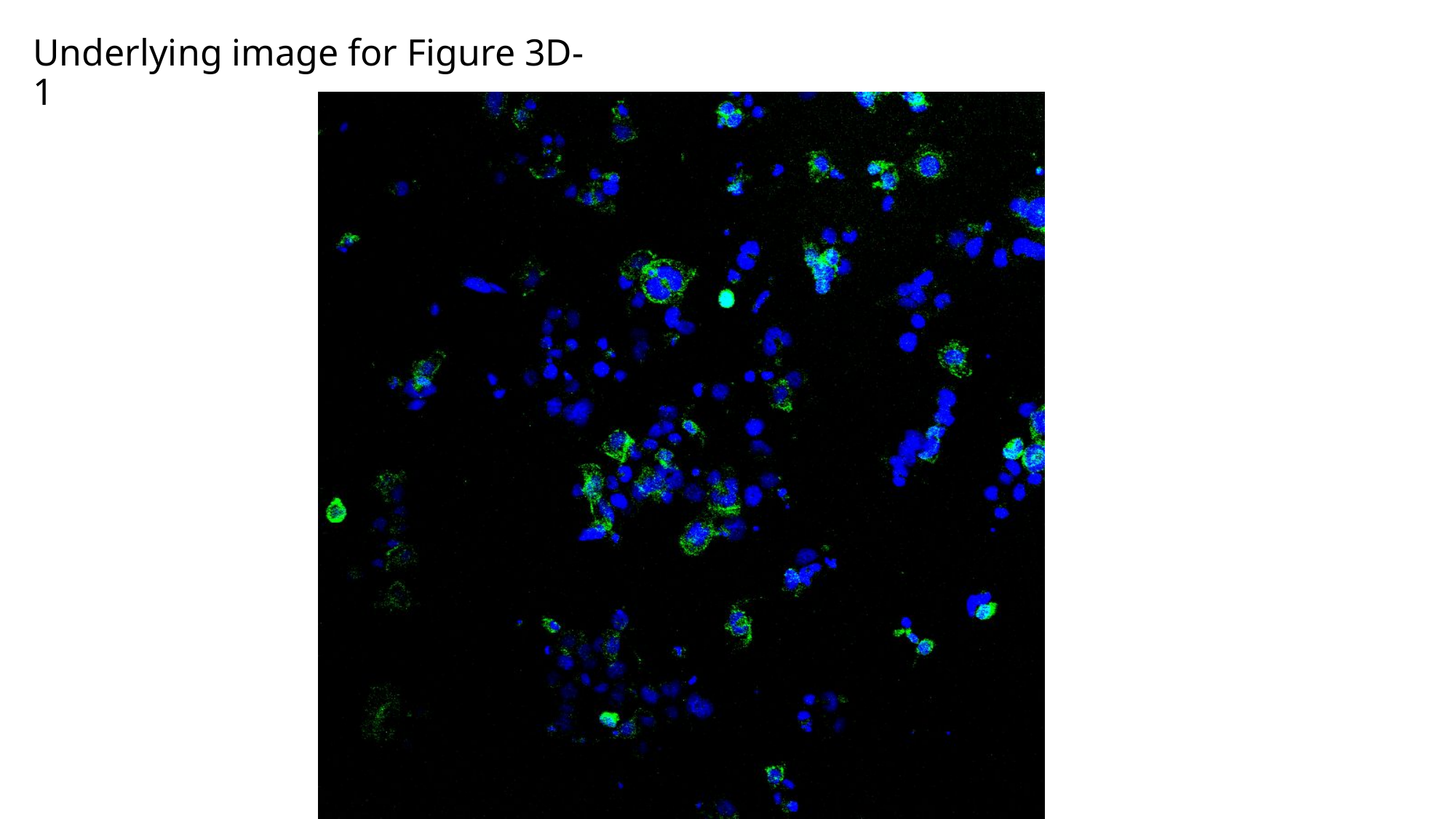

# Underlying image for Figure 3D-1

## Slide 4
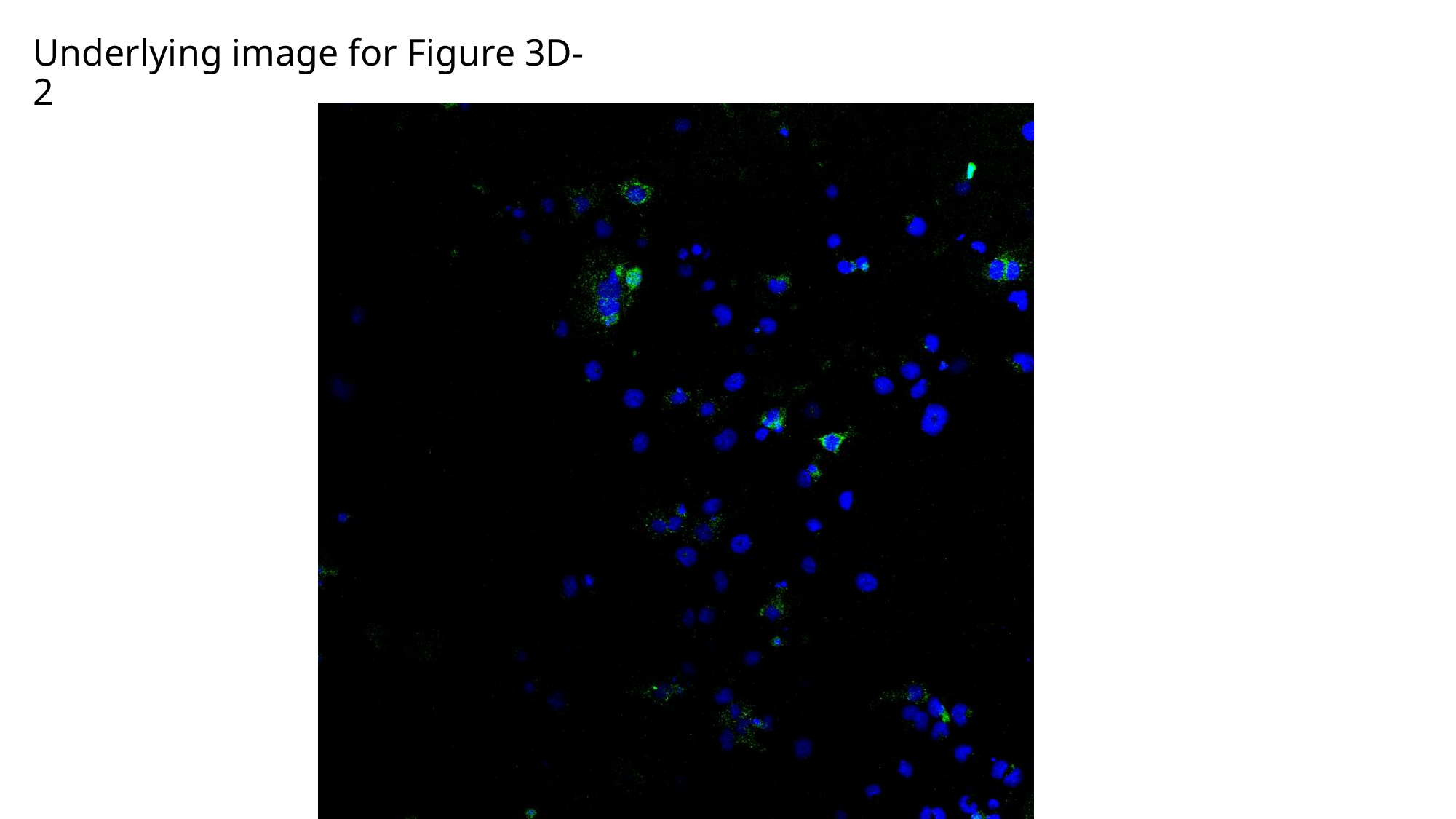

# Underlying image for Figure 3D-2

## Slide 5
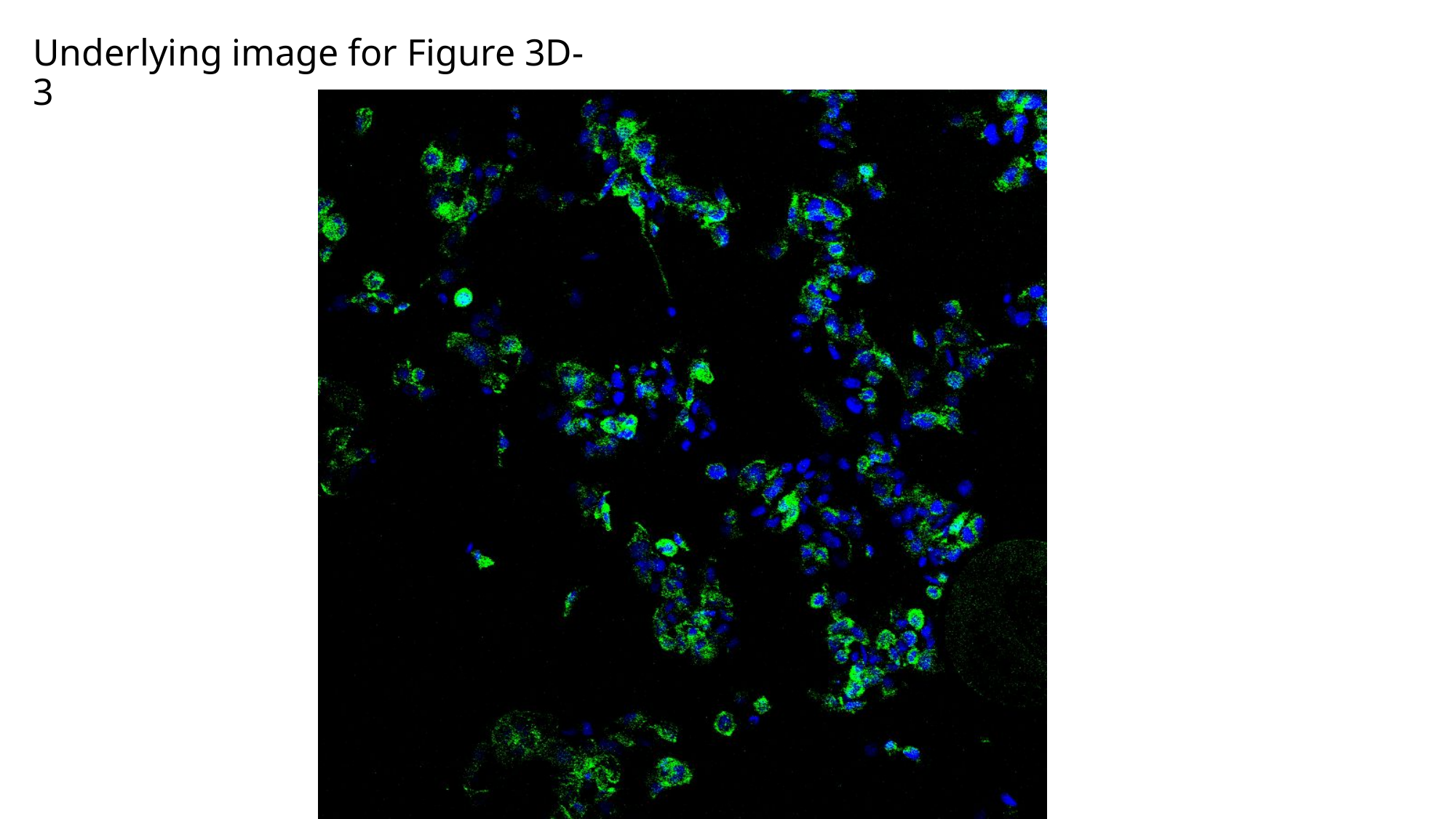

# Underlying image for Figure 3D-3

## Slide 6
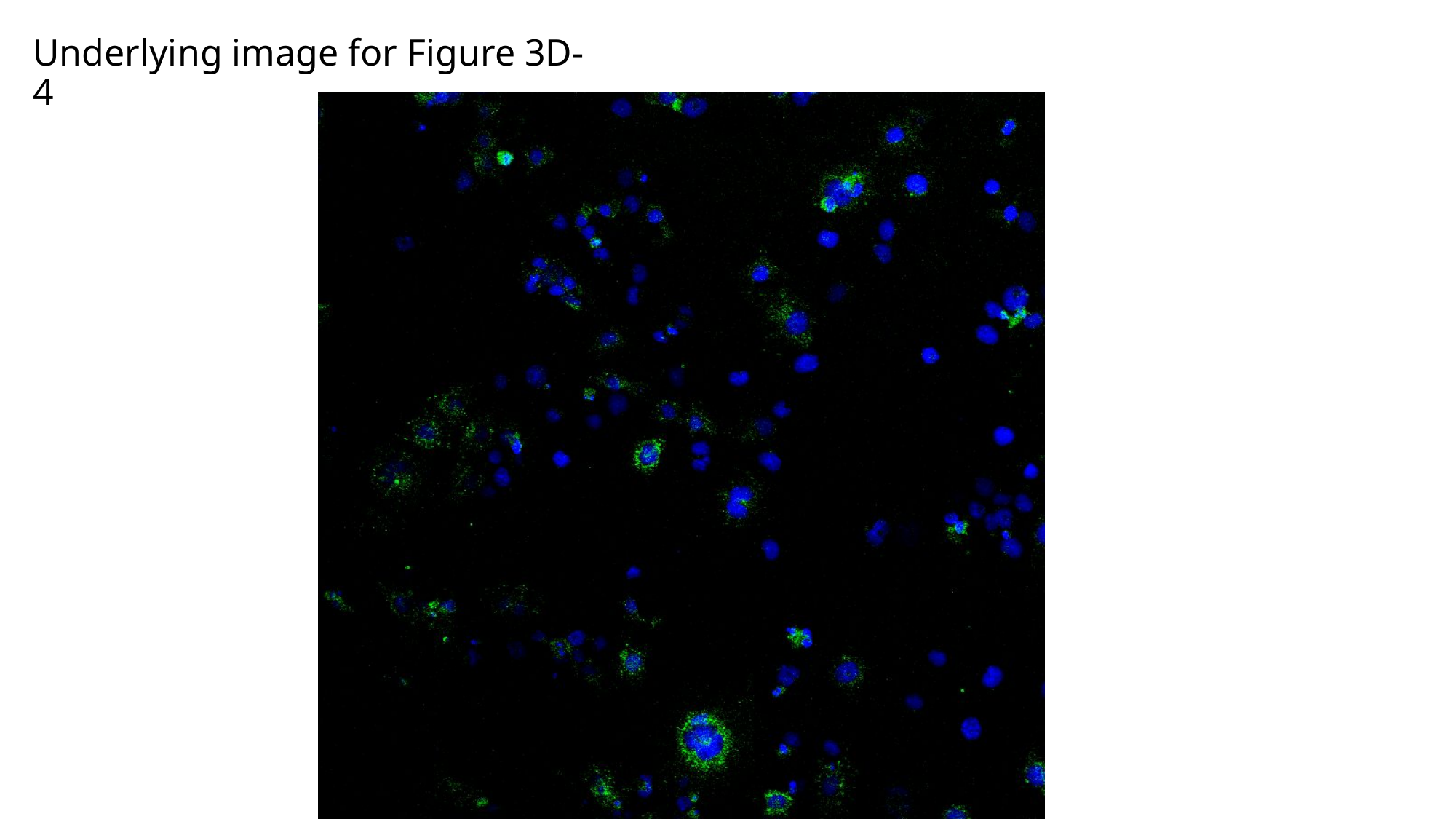

# Underlying image for Figure 3D-4

## Slide 7
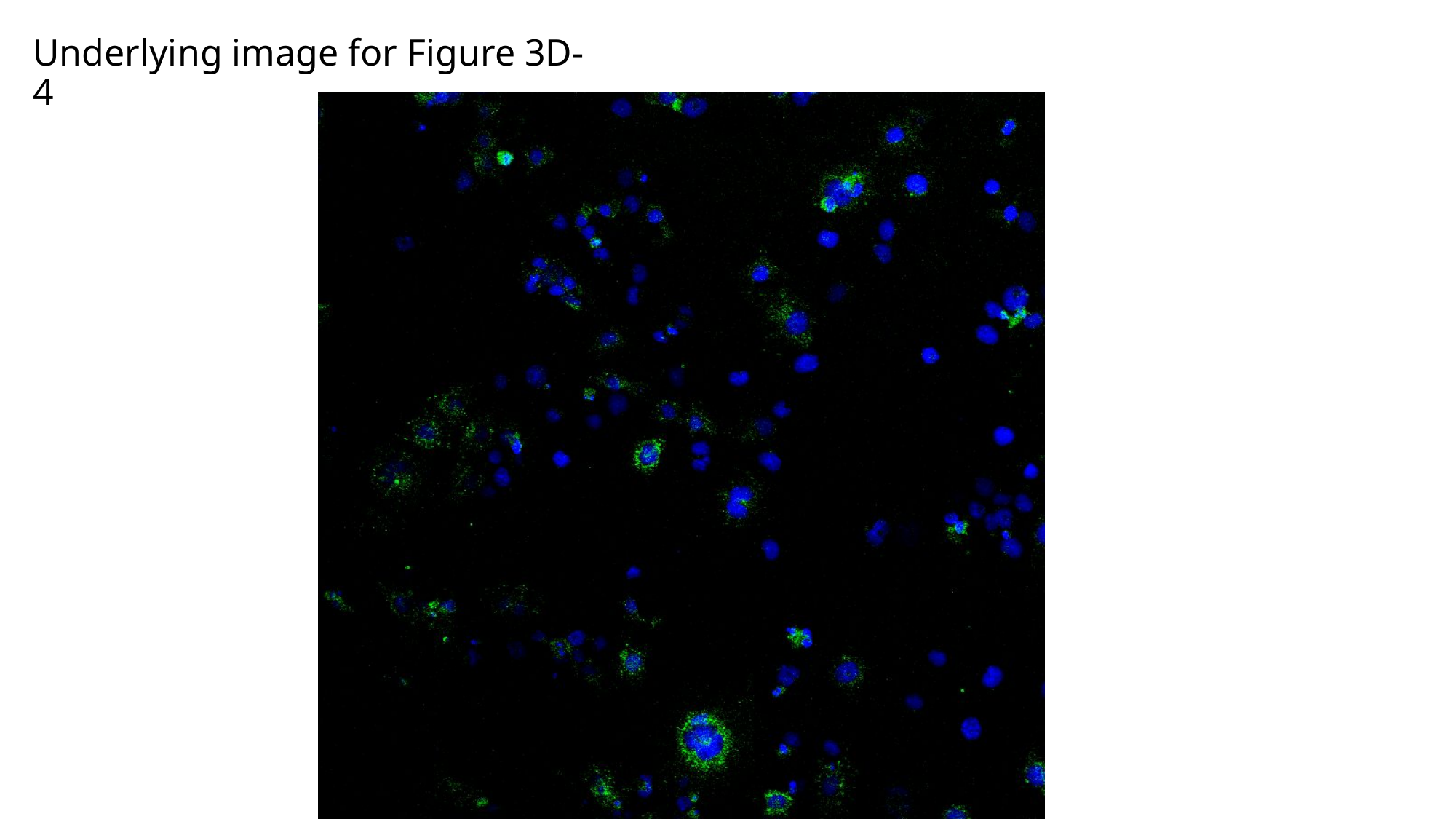

# Underlying image for Figure 3D-4

## Slide 8
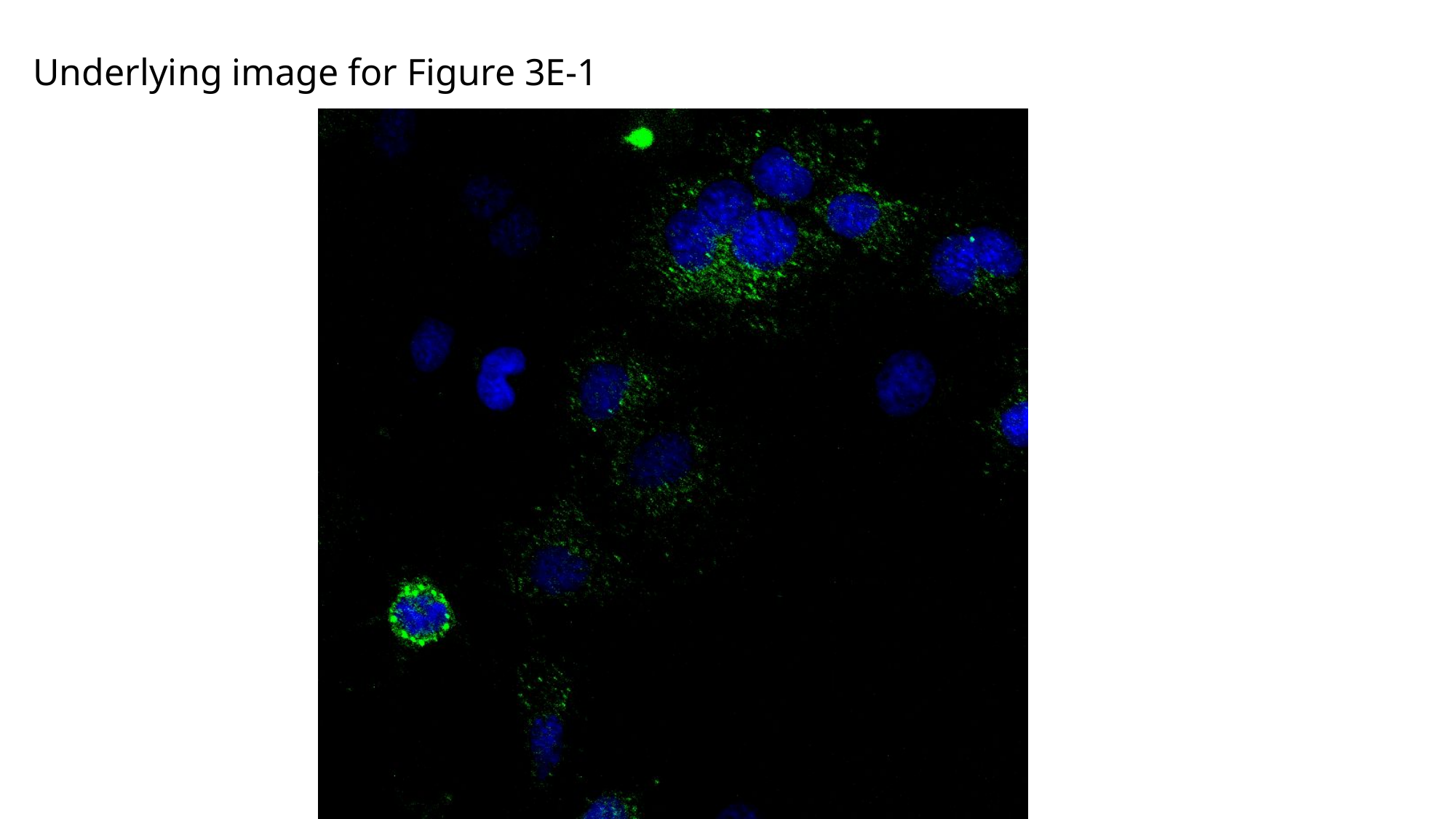

# Underlying image for Figure 3E-1

## Slide 9
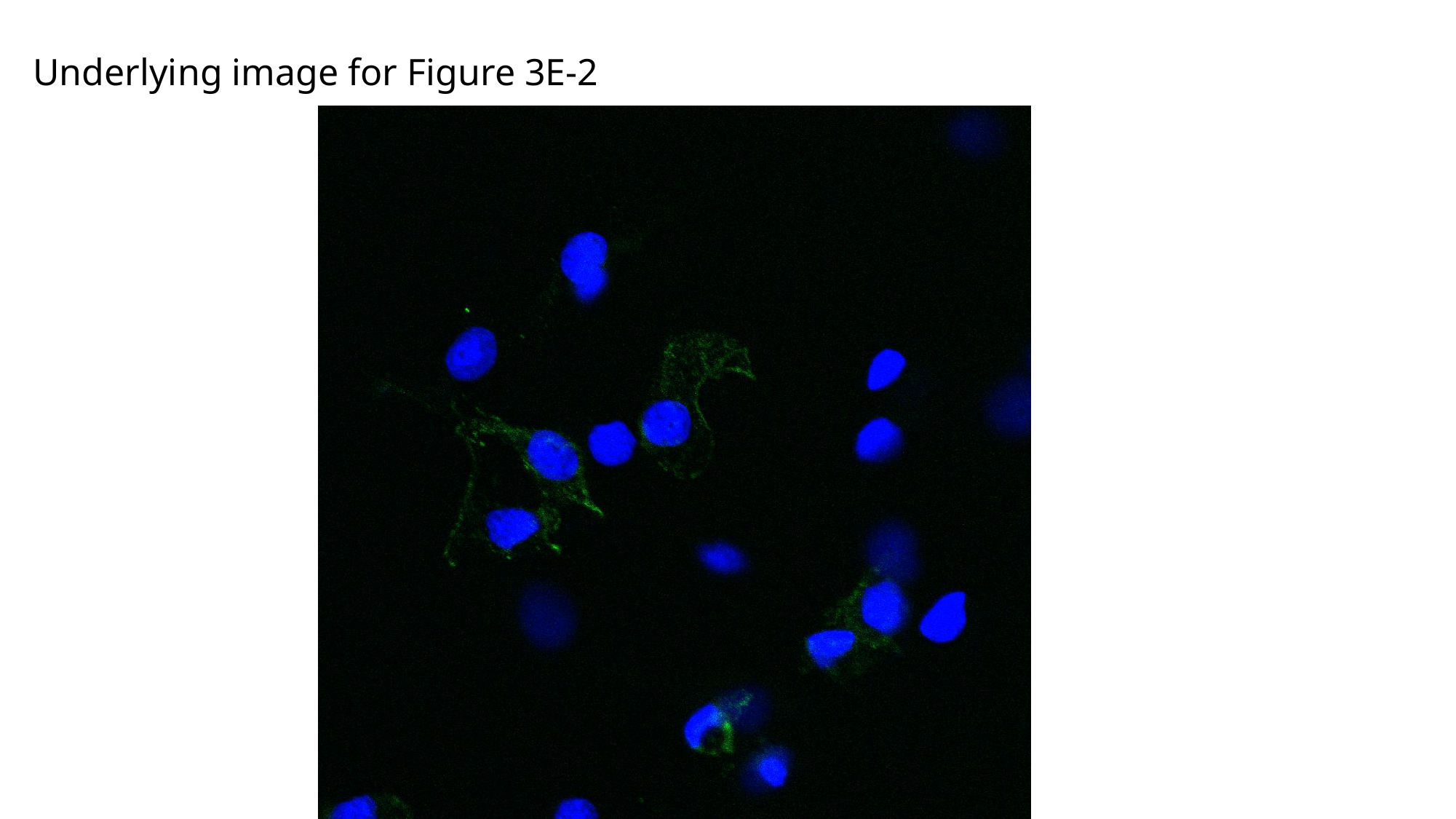

# Underlying image for Figure 3E-2

## Slide 10
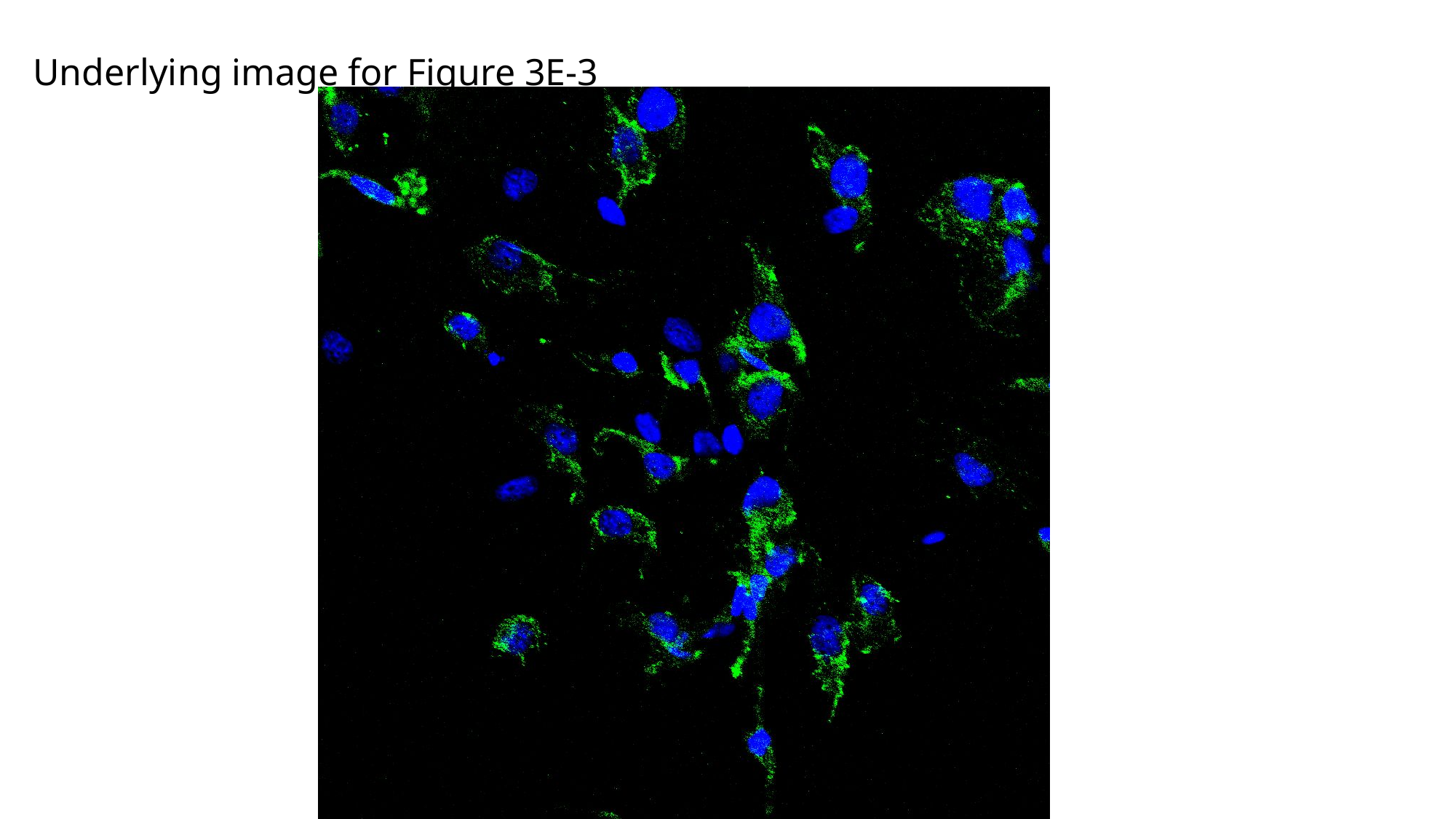

# Underlying image for Figure 3E-3

## Slide 11
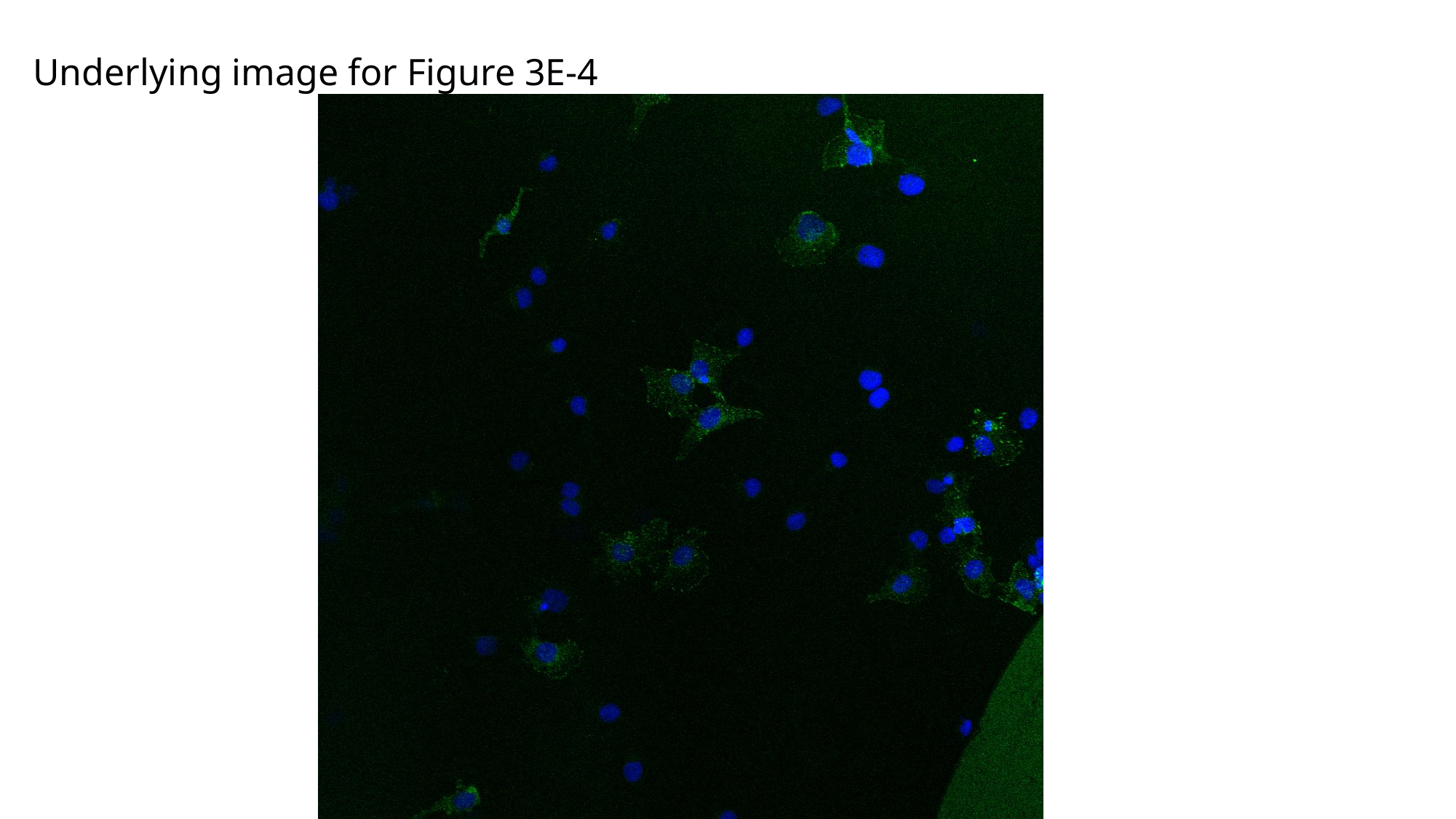

# Underlying image for Figure 3E-4

## Slide 12
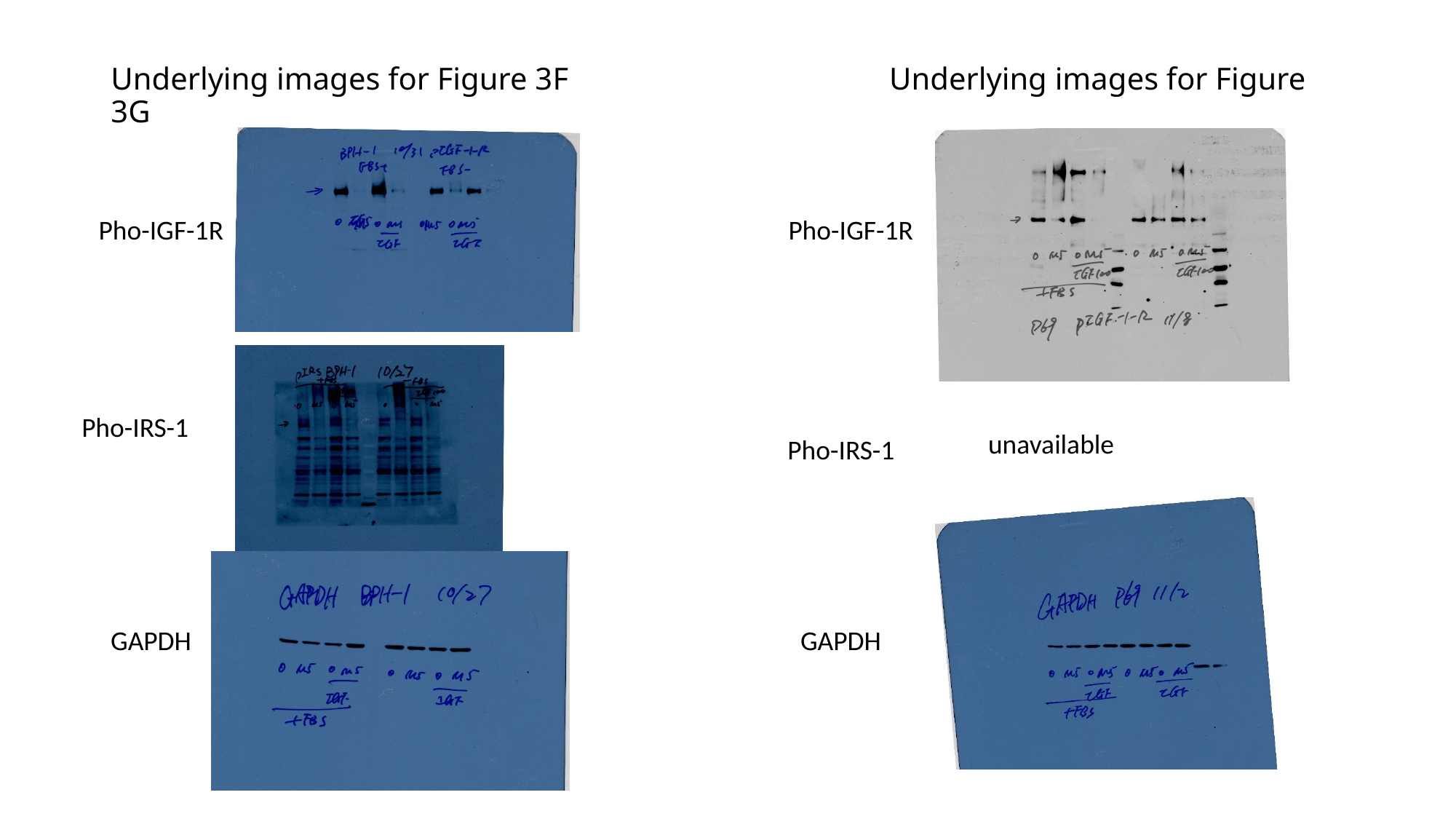

# Underlying images for Figure 3F Underlying images for Figure 3G
Pho-IGF-1R
Pho-IGF-1R
Pho-IRS-1
unavailable
Pho-IRS-1
GAPDH
GAPDH
