# Supplementary figures and images for: Correction: Metformin inhibits the proliferation of benign prostatic epithelial cells
Source: PLoS One. 2023 Dec 12;18(12):e0295893. doi: 10.1371/journal.pone.0295893 (PMC10715638; doi:10.1371/journal.pone.0295893)

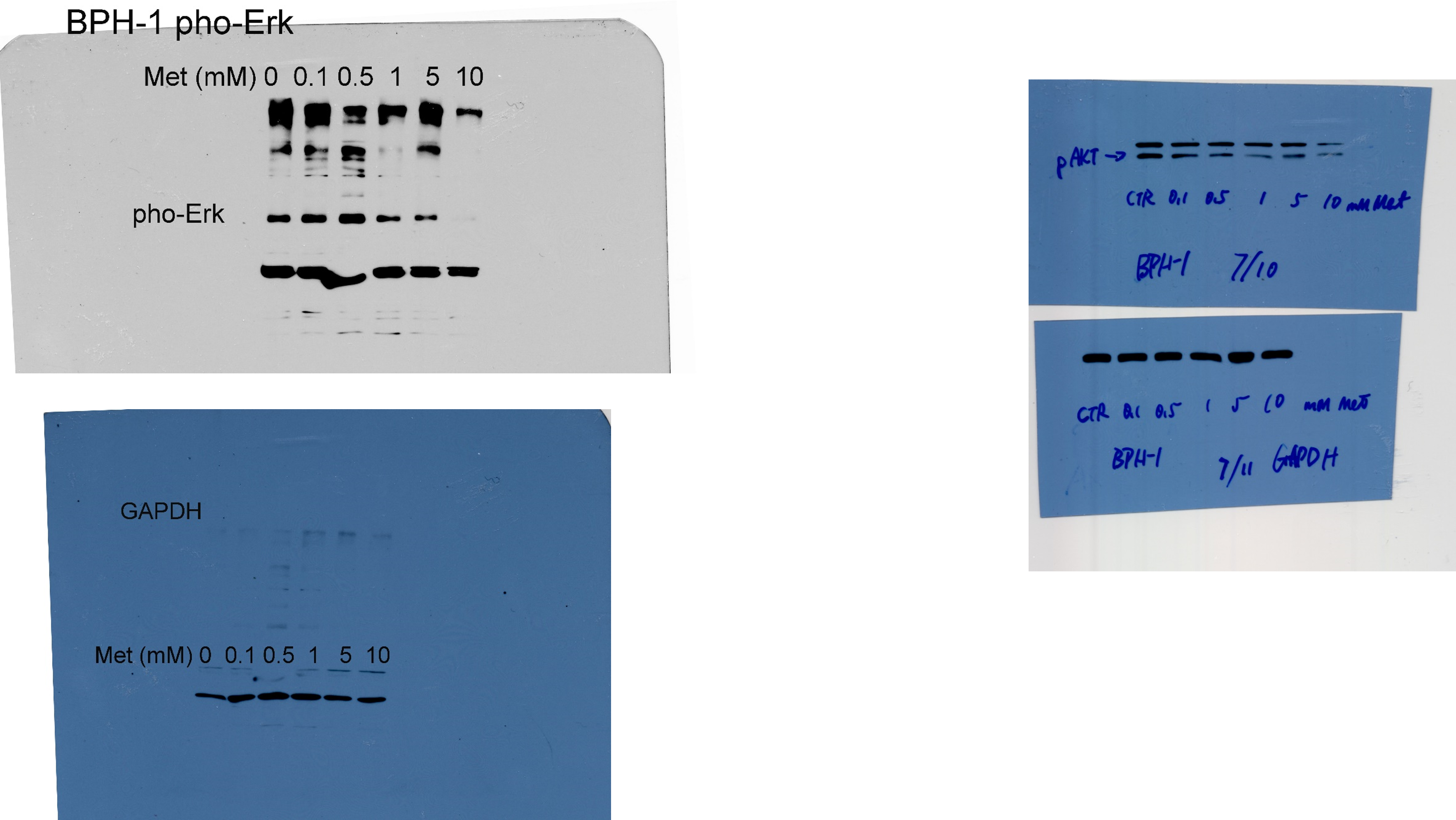

Supplement: S4 File — (TIF) [file pone.0295893.s004.tif]
